# Clinical Drivers of Cesarean Sections During the COVID-19 Pandemic: A Robson Classification-Based Multivariable Analysis

**DOI:** 10.7759/cureus.113347

**Published:** 2026-07-25

**Authors:** Sreelakshme Nilakandan, Pavithra Devi Babu, Kiruba Haridassan, Thamizhmaran Sundararajan

**Affiliations:** 1 Obstetrics and Gynaecology, Pondicherry Institute of Medical Sciences, Puducherry, IND; 2 Community Medicine, Mahatma Gandhi Medical College and Research Institute, Sri Balaji Vidyapeeth University, Puducherry, IND

**Keywords:** cesarean section, covid-19 pandemic, high-risk pregnancy, maternal morbidity, multivariable analysis, obstetric triage, robson classification

## Abstract

Background and objective: The COVID-19 pandemic severely disrupted global maternity care, prompting concerns that logistical pressures and infection risks lowered the clinical threshold for performing cesarean sections (LSCS). This study aimed to determine whether delivering during the pandemic epoch independently increased a patient's odds of undergoing a surgical delivery, adjusting for pandemic-induced demographic shifts using the WHO Robson Ten Group Classification System.

Methods: A retrospective cohort study of 3,656 deliveries was conducted at a tertiary care center, comparing pre-pandemic (n = 2,819) and pandemic (n = 837) epochs. Baseline demographics, antenatal comorbidities, and delivery outcomes were analyzed. A multivariable binomial logistic regression model was constructed to isolate the independent effect of the pandemic on delivery mode, adjusting for maternal age, comorbidities, and Robson mega-groups.

Results: The pandemic induced a significant "triage effect," concentrating high-risk admissions. The pandemic cohort was significantly older (p < 0.001) and possessed a higher burden of metabolic comorbidities, including gestational diabetes mellitus (38.2% vs. 26.6%, p < 0.001). Despite this increased clinical complexity, the overall crude LSCS rate remained stable (33.5% vs. 34.8%, p = 0.518). Multivariable regression confirmed that the pandemic epoch did not independently increase the odds of a surgical delivery (aOR = 0.942; 95% CI: 0.768-1.156; p = 0.567). Surgical intervention was overwhelmingly driven by established obstetric indications, notably previous uterine scars (Robson 5; aOR = 22.45), malpresentations or multiple gestation (Robson 6-9; aOR = 10.70), and severe preeclampsia (aOR = 3.05). Unadjusted associations between metabolic comorbidities and LSCS lost significance in the adjusted model, proving them to be confounding variables mediated by age and prior uterine scars.

Conclusion: While the COVID-19 lockdown fundamentally shifted the demographic profile of obstetric admissions toward a highly complex, comorbid population, evidence-based surgical thresholds were rigorously maintained. Institutional increases in surgical urgency were a direct mathematical reflection of the triaged patient profile rather than a degradation of clinical practice standards.

## Introduction

The COVID-19 pandemic precipitated an unprecedented disruption of global healthcare systems, fundamentally altering the landscape of maternity care. To mitigate the spread of the SARS-CoV-2 virus, sweeping lockdowns were implemented, healthcare resources were reallocated, and strict infection control protocols were enforced. Consequently, obstetric care-seeking behaviors shifted dramatically. Fear of nosocomial infection, compounded by severe mobility restrictions, led many low-risk pregnant women to bypass large metropolitan hospitals in favor of peripheral midwifery-led units, smaller local clinics, or home births [1]. In contrast, tertiary care facilities were rapidly transformed into high-acuity triage hubs, absorbing a heavily concentrated influx of complicated and high-risk pregnancies [2,3].

During the initial waves of the pandemic, this sudden reorganization of patient volume coincided with alarming reports in the global literature detailing sharp spikes in institutional cesarean section rates [4]. Existing studies have raised clinical and ethical concerns regarding whether the severe logistical constraints of the pandemic - including acute staffing shortages, resource rationing, and the deliberate strategy to minimize healthcare provider exposure during prolonged vaginal labors - inadvertently lowered the clinical threshold for surgical deliveries [1,5].

The World Health Organization (WHO) had recommended Robson Ten Group Classification System serves as the global gold standard for assessing, comparing, and monitoring cesarean delivery rates [6]. By categorizing women into mutually exclusive groups based on fundamental obstetric characteristics, parity, previous uterine scars, gestational age, onset of labor, and fetal presentation, the Robson classification effectively mitigates the confounding variables that plague crude data comparisons [7].

This study utilized the Robson classification framework to investigate the core clinical drivers of caesarean sections during the lockdown period. Specifically, we sought to determine if delivering during the COVID-19 pandemic independently elevated a patient's odds of undergoing a caesarean delivery.

## Materials and methods

Study design and setting

A retrospective, single-center cohort study was conducted at a tertiary care teaching hospital in Puducherry. The study evaluated obstetric admissions over a 48-month period, encompassing a pre-pandemic epoch, January 2018 to February 2020, and a COVID-19 pandemic epoch, March 2020 to December 2021. The study protocol was reviewed and approved by the Institutional Ethics Committee (IEC number - RC/2024/09), and the requirement for informed patient consent was waived due to the retrospective nature of the de-identified data analysis.

Study population

All pregnant women who delivered a viable fetus (≥28 weeks of gestation) at the study institution during the defined 48-month timeframe were eligible for inclusion. Cases with incomplete or missing primary outcome data (mode of delivery or gestational age) were excluded from the final analysis. A total of 3,656 deliveries met the inclusion criteria, yielding a pre-pandemic cohort of 2,819 patients and a pandemic cohort of 837 patients.

Data collection and variable definitions

De-identified clinical data were systematically extracted from the hospital's electronic and physical obstetric medical records. The extracted variables included maternal demographics (age, gravidity, parity, and obstetric history), gestational age at delivery, labor characteristics (onset of labor, fetal presentation), and primary mode of delivery (vaginal delivery, elective lower segment cesarean section (LSCS), or emergency LSCS).

Maternal comorbidities and labor complications were standardized from raw clinical text into binary variables. Key clinical covariates included gestational diabetes mellitus (GDM), maternal obesity, thyroid disorders (clinical and subclinical hypothyroidism), chronic or gestational hypertension, severe preeclampsia/eclampsia, and bad obstetric history (BOH). Acute intrapartum surgical indications, such as fetal distress, cephalopelvic disproportion (CPD), and cord prolapse, were also recorded.

Robson Ten Group Classification System

To objectively compare cesarean rates and adjust for the shifting baseline characteristics of the study cohorts, all patients were categorized according to the WHO Robson Ten Group Classification System [8]. To ensure statistical stability for multivariable modeling and to prevent sample size fragmentation, the 10 standard Robson groups were consolidated into five robust clinical mega-groups based on established obstetric risk profiles:

Robson 1 & 2: Low-risk, nulliparous women with a single cephalic pregnancy at term (the standard reference population).

Robson 3 & 4: Multiparous women (without a previous uterine scar) with a single cephalic pregnancy at term.

Robson 5: All women with at least one previous uterine section, single cephalic pregnancy at term.

Robson 6-9: Absolute or high-risk indications, including all multiple gestations, breech presentation, abnormal fetal lies (transverse, oblique), and abnormal lies with previous scars.

Robson 10: All women with a single cephalic pregnancy resulting in a preterm delivery (<37 weeks of gestation).

Statistical analysis

Statistical analyses were performed using R software (version 4.2.3; R Development Core Team, Vienna, Austria). Continuous variables, such as maternal age, were assessed for normality and presented as means with standard deviations (± SD); comparisons between the pre-pandemic and pandemic cohorts were conducted using independent Student's t-tests. Categorical variables were presented as frequencies and percentages, and group differences were analyzed using Pearson's chi-square test.

To identify independent predictors of the primary outcome (delivery mode: LSCS vs. NVD), both unadjusted and adjusted binomial logistic regression models were constructed. The multivariable regression model incorporated time period (pandemic vs. pre-pandemic), maternal age, antenatal comorbidities, and the categorized Robson mega-groups as independent covariates. The results of the regression analyses were reported as unadjusted odds ratios (OR) and adjusted odds ratios (aOR) with 95% confidence intervals (CI). Overall model fit was evaluated using the omnibus Chi-square test and Nagelkerke's pseudo-R2. For all analyses, a two-tailed p-value of < 0.05 was considered statistically significant.

## Results

Baseline demographic and clinical shifts

A total of 3,656 deliveries met the inclusion criteria (2,819 pre-pandemic; 837 pandemic). The onset of the pandemic was associated with a significant demographic shift toward high-risk pregnancies (Table 1). The pandemic cohort was significantly older (p < 0.001) and comprised a higher proportion of multiparous women (p = 0.002) and preterm deliveries (14.7% (n = 123) vs. 11.8% (n = 332), p < 0.001). Furthermore, the burden of antenatal comorbidities intensified markedly; 82.1% (n = 687) of the pandemic cohort presented with at least one complication compared to 74.7% (n = 2,106) pre-pandemic (p < 0.001). Most notably, the prevalence of GDM (38.2% (n = 320) vs. 26.6% (n = 751), p < 0.001), maternal obesity (11.2% (n = 94) vs. 4.4% (n = 125), p < 0.001), and thyroid disorders (25.7 (n = 215) % vs. 21.2% (n = 215), p = 0.007) spiked during the lockdown epoch.

**Table 1:** Baseline demographic and obstetric characteristics Age (years) - An independent t-test was applied. Categorial variables: Chi-square test was applied. LSCS, lower segment cesarean section; NVD, normal vaginal delivery; IUGR, intrauterine growth restriction; FGR, fetal growth restriction; PPROM, preterm premature rupture of membranes; PROM, premature rupture of membranes; GDM, gestational diabetes mellitus

Baseline Demographic and Obstetric Characteristics	Pre-pandemic (n=2819)	Pandemic (n=837)	Total (N=3656)	Test Statistic (t/χ²)	p
Age (years), Mean (SD)	25.9 (4.4)	27.4 (4.4)	26.3 (4.4)	8.32	<0.001
Gravida Status					
Grand multigravida	56 (2.0)	9 (1.1)	65 (1.8)	32.04	<0.001
Multigravida	1385 (49.1)	503 (60.1)	1888 (51.6)		
Primi	1378 (48.9)	325 (38.8)	1703 (46.6)		
Parity Status					
Multipara	211 (7.5)	57 (6.8)	268 (7.3)	17.67	<0.001
Nullipara	1591 (56.4)	411 (49.1)	2002 (54.8)		
Primipara	1017 (36.1)	369 (44.1)	1386 (37.9)		
Live Children Status					
L0	1627 (57.7)	418 (49.9)	2045 (55.9)		<0.001
L1	1021 (36.2)	376 (44.9)	1397 (38.2)	20.99	
L2	164 (5.8)	42 (5.0)	206 (5.6)		
L>/=3	7 (0.2)	1 (0.1)	8 (0.2)		
Abortion Status					
Nulliabortus	2247 (79.7)	631 (75.4)	2878 (78.7)	7.97	0.047
Primiabortus	431 (15.3)	161 (19.2)	592 (16.2)		
A2	101 (3.6)	33 (3.9)	134 (3.7)		
A3 or more	40 (1.4)	12 (1.4)	52 (1.4)		
Single/Multiple-Ton Pregnancy					
Multiple	69 (2.4)	27 (3.2)	96 (2.6)	1.528	0.216
Single	2750 (97.6)	810 (96.8)	3560 (97.4)		
Pre/Post/Term					
Post-term	306 (10.9)	43 (5.1)	349 (9.5)		<0.001
Preterm	332 (11.8)	123 (14.7)	455 (12.4)	27.17	
Term	2181 (77.4)	671 (80.2)	2852 (78.0)		
Gestation Age at Delivery					
Early preterm	45 (1.6)	14 (1.7)	59 (1.6)		<0.001
Late preterm	239 (8.5)	92 (11.0)	331 (9.1)	27.67	
Post-term	306 (10.9)	43 (5.1)	349 (9.5)		
Preterm	48 (1.7)	17 (2.0)	65 (1.8)		
Term	2181 (77.4)	671 (80.2)	2852 (78.0)		
Antenatal comorbidities	2106 (74.7)	687 (82.1)	2793 (76.4)	19.45	<0.001
GDM	751 (26.6)	320 (38.2)	1071 (29.3)	41.86	<0.001
Hypertensive disorders (GHTN or chronic HTN)	157 (5.6)	54 (6.5)	211 (5.8)	0.924	0.336
Preeclampsia & eclampsia	65 (2.3)	15 (1.8)	80 (2.2)	0.796	0.372
Hypothyroidism	598 (21.2)	215 (25.7)	813 (22.2)	7.470	0.006
Anemia	257 (9.1)	36 (4.3)	293 (8.0)	20.302	<0.001
Fetal growth restriction	156 (5.5)	36 (4.3)	192 (5.3)	1.971	0.160
Oligohydramnios	162 (5.7)	19 (2.3)	181 (5.0)	16.578	<0.001
Polyhydramnios	17 (0.6)	11 (1.3)	28 (0.8)	4.295	0.038
Membrane rupture (PROM/PPROM)	230 (8.2)	77 (9.2)	307 (8.4)	0.909	0.341
Obesity	125 (4.4)	94 (11.2)	219 (6.0)	52.937	<0.001
Bad obstetric history	41 (1.5)	11 (1.3)	52 (1.4)	0.090	0.764
Rh-negative	149 (5.3)	40 (4.8)	189 (5.2)	0.338	0.561
Previous LSCS	381 (13.5)	130 (15.5)	511 (14.0)	2.182	0.140
Labour Characteristics					
Onset of Labour					
Spontaneous	1284 (45.5)	374 (44.7)	1658 (45.4)	0.315	0.854
Induced	942 (33.4)	280 (33.5)	1222 (33.4)		
Prelabour Cs	593 (21.0)	183 (21.9)	776 (21.2)		
Mode of Delivery					
NVD	1875 (66.5)	546 (65.2)	2421 (66.2)	15.574	<0.001
Elective LSCS	261 (9.3)	115 (13.7)	376 (10.3)		
Emergency LSCS	683 (24.2)	176 (21.0)	859 (23.5)		
Previous LSCS	263 (9.3)	78 (9.3)	341 (9.3)	0	0.993
Complications During Delivery/Labour That Indicated LSCS					
Fetal distress	206 (7.3)	36 (4.3)	242 (6.6)	9.438	0.002
Malpresentation	92 (3.3)	28 (3.3)	120 (3.3)	0.014	0.907
Cephalopelvic disproportion (CPD) & contracted pelvis	143 (5.1)	48 (5.7)	191 (5.2)	0.571	0.450
Failure to progress/arrest of labor	16 (0.6)	10 (1.2)	26 (0.7)	3.595	0.058
Meconium-stained liquor	3 (0.1)	0 (0.0)	3 (0.1)	0.066	0.797
Placental complications	33 (1.2)	10 (1.2)	43 (1.2)	0.003	0.955
Hypertensive disorders (preeclampsia, eclampsia)	21 (0.7)	12 (1.4)	33 (0.9)	3.423	0.064
Multiple gestation	34 (1.2)	15 (1.8)	49 (1.3)	1.676	0.195
IUGR/FGR	9 (0.3)	2 (0.2)	11 (0.3)	0.139	0.710
Membrane issues (PROM, PPROM)	30 (1.1)	4 (0.5)	34 (0.9)	2.408	0.121
Cord complications (prolapse, presentation)	5 (0.2)	1 (0.1)	6 (0.2)	0.132	0.176
Robson Group					
1	605 (21.5)	137 (16.4)	742 (20.3)	30.26	<0.001
2	739 (26.2)	185 (22.1)	924 (25.3)		
3	466 (16.5)	162 (19.4)	628 (17.2)		
4	272 (9.6)	106 (12.7)	378 (10.3)		
5	304 (10.8)	109 (13.0)	413 (11.3)		
6	49 (1.7)	9 (1.1)	58 (1.6)		
7	39 (1.4)	8 (1.0)	47 (1.3)		
8	69 (2.4)	28 (3.3)	97 (2.7)		
9	14 (0.5)	3 (0.4)	17 (0.5)		
10	262 (9.3)	89 (10.6)	351 (9.6)		
Robson Group Category					
Robson 1&2	1344 (47.7)	322 (38.5)	1666 (45.6)		<0.001
Robson 3&4	738 (26.2)	268 (32.1)	1006 (27.5)		
Robson 5	304 (10.8)	109 (13.0)	413 (11.3)	24.20	
Robson 6-9	171 (6.1)	48 (5.7)	219 (6.0)		
Robson 10	262 (9.3)	89 (10.6)	351 (9.6)		

Delivery modes and surgical indications

Despite the significantly higher clinical complexity of the pandemic cohort, the overall binary rate of cesarean delivery remained statistically stable between the two epochs (33.8% overall LSCS; p = 0.518). However, the urgency and indications for surgical intervention shifted significantly. While the onset of labor (spontaneous vs. induced) remained unchanged (p = 0.854), there was a marked transition from emergency to elective procedures (p < 0.001). Elective LSCS procedures increased significantly during the pandemic (13.7% (n = 115) vs. 9.3% (n = 261)), whereas emergency LSCS procedures decreased (21.0% (n = 176) vs. 24.2% (n = 24.2)). Correspondingly, intrapartum fetal distress as a primary surgical indication dropped significantly during the lockdown months (4.3% (n = 36) vs. 7.3% (n = 206); p = 0.003). Obstetric indications for surgery, such as cephalopelvic disproportion (5.2% (n = 191) overall, p = 0.505) and malpresentation (3.3% (n = 120) overall, p = 0.995), showed no significant variation between the two time periods.

Robson Ten Group Classification System dynamics

The changing clinical profile of the hospital was most clearly reflected in the shifting distribution of the Robson classification groups (p < 0.001). The proportion of standard, low-risk nulliparous women with single cephalic term pregnancies (Robson Groups 1 & 2), the primary benchmark population for evaluating a facility's baseline obstetric care, contracted significantly from 47.7% (n = 1,344) in the pre-pandemic period to 38.5% (n = 322) during the pandemic.

In contrast, the facility absorbed a significantly higher proportion of patients with established obstetric histories. Deliveries among low-risk multiparous women (Robson Groups 3 & 4) increased from 26.2% (n = 738) to 32.1% (n = 268). Most notably, women presenting with a previous cesarean scar (Robson Group 5), a primary biological driver of repeat surgical deliveries, rose from 10.8% (n = 304) to 13.0% (n = 109) of the hospital's total volume. Finally, the proportion of preterm deliveries (Robson Group 10) increased (9.3% (n = 1344) vs 10.6% (n = 89)), aligning with the higher maternal comorbidity burden and increased elective surgical triage observed during the lockdown months.

Unadjusted and multivariable predictors of cesarean delivery

To determine the independent predictors of cesarean delivery, both unadjusted and adjusted binomial logistic regression models were analyzed (Table 2). The multivariable model was constructed to account for the significant demographic and clinical shifts observed during the pandemic, incorporating maternal age, relevant antenatal comorbidities, and the grouped Robson classification as covariates.

**Table 2:** Multivariable logistic regression to determine predictors of cesarean delivery LSCS, lower segment cesarean section; NVD, normal vaginal delivery; GDM, gestational diabetes mellitus; GHTN, gestational hypertension; HTN, hypertension

Parameters	LSCS (N=1235)	NVD (N=2421)	Unadjusted OR (95% CI)	p-value	Adjusted OR (95% CI)	p-value
Time Period						
Pandemic	291 (23.6)	546 (22.6)	1.06 (0.90-1.25)	0.492	0.942 (0.768-1.156)	0.567
Pre-pandemic	944 (76.4)	1875 (77.4)				
Age Mean (SD)	27.2 (4.7)	25.8 (4.2)	1.43 (1.13-1.73)	<0.001	1.073 (1.052-1.096)	<0.001
GDM						
Present	421 (34.1)	650 (26.8)	1.41 (1.22-1.63)	<0.001	1.138 (0.945-1.371)	0.174
Absent	814 (65.9)	1771 (73.2)				
Hypertensive Disorders (GHTN or Chronic HTN)						
Present	91 (7.4)	120 (5.0)	1.52 (1.15-2.02)	0.003	1.305 (0.934-1.824)	0.119
Absent	1144 (92.6)	2301 (95.0)				
Preeclampsia & Eclampsia						
Present	53 (4.3)	27 (1.1)	3.97 (2.5-6.42)	<0.001	3.053 (1.792-5.203)	<0.001
Absent	1182 (95.7)	2394 (98.9)				
Thyroid Issues (Hypothyroidism and Subclinical Hypothyroidism)						
Present	304 (24.6)	509 (21.0)	1.23 (1.04-1.44)	0.014	1.136 (0.932-1.385)	0.207
Absent	931 (75.4)	1912 (79.0)				
Bad Obstetric History						
Present	27 (2.2)	25 (1.0)	2.14 (1.23-3.73)	0.005	2.275 (1.12-4.622)	0.023
Absent	1208 (97.8)	2396 (99.0)				
Robson Group						
Robson 1&2	432 (35.0)	1234 (51.0)	1			
Robson 3&4	91 (7.4)	915 (37.8)	0.28 (0.22-0.36)	<0.001	0.23 (0.178-0.296)	<0.001
Robson 5	373 (30.2)	40 (1.7)	26.59 (19.01-37.92)	<0.001	22.45 (15.847-31.803)	<0.001
Robson 6-9	178 (14.4)	41 (1.7)	12.38 (8.73-17.86)	<0.001	10.701 (7.452-15.369)	<0.001
Robson 10	160 (13.0)	191 (7.9)	2.39 (1.89-3.03)	<0.001	1.903 (1.485-2.438)	<0.001

The regression analysis confirmed that delivering during the COVID-19 pandemic epoch did not increase a patient's odds of undergoing a cesarean section. This remained true in both the crude, unadjusted analysis (OR = 1.06; 95% CI: 0.90-1.25; p = 0.492) and the final multivariable model (aOR = 0.942; 95% CI: 0.768-1.156; p = 0.567).

The analysis revealed a profound confounding effect regarding maternal metabolic comorbidities. In the unadjusted analysis, GDM (OR = 1.41, p < 0.001), hypertensive disorders (OR = 1.52, p = 0.003), and thyroid issues (OR = 1.23, p = 0.014) were all significantly associated with higher odds of surgical delivery. However, after mathematically adjusting for maternal age and obstetric history, all three of these conditions lost their statistical significance (p > 0.05). This demonstrates that their apparent influence on the delivery mode was entirely mediated by advancing maternal age and previous surgical scars, rather than the metabolic conditions acting as independent triggers for surgery.

The true, independent drivers of surgical delivery were rooted in severe maternal morbidity and established obstetric history. Preeclampsia and eclampsia remained a massive independent risk factor, tripling the odds of an LSCS (aOR = 3.05; p < 0.001), while advancing maternal age increased the odds by 7.3% per year (aOR = 1.07; p < 0.001). A BOH also significantly more than doubled the odds of surgical intervention (aOR = 2.27; p = 0.023).

Finally, the Robson classification served as the strongest predictor of delivery mode. Using low-risk, term nulliparous women (Robson Groups 1 & 2) as the reference baseline, the odds of LSCS were overwhelmingly driven by previous cesarean scars (Robson 5; aOR = 22.45, p < 0.001) and absolute surgical indications, such as malpresentations and multiple gestations (Robson 6-9; aOR = 10.70, p < 0.001). Preterm deliveries (Robson 10) also independently nearly doubled the odds of surgery (aOR = 1.90; p < 0.001), while low-risk multiparous women (Robson 3 & 4) exhibited a highly significant 77% reduction in surgical odds (aOR = 0.23; p < 0.001).

## Discussion

The primary objective of this study was to determine whether the logistical and clinical pressures of the COVID-19 pandemic altered the threshold for performing cesarean deliveries. Our findings demonstrate that, while the pandemic caused a profound demographic shift toward older, multiparous, and highly comorbid admissions, delivering during the lockdown epoch did not independently increase a patient's odds of undergoing a surgical delivery (aOR = 0.942). Furthermore, while crude, unadjusted analyses suggested that metabolic conditions, such as GDM and hypertensive disorders, significantly increased surgical risk, multivariable adjustments revealed these to be confounding variables. Their influence was heavily mediated by advancing maternal age and prior uterine scars. Ultimately, clinical decision-making remained resolute; surgical interventions were driven entirely by established, absolute obstetric indications rather than pandemic-induced logistical pressures.

The profound baseline demographic shifts observed within our cohort - specifically the significant contraction of low-risk, term nulliparous admissions (Robson Groups 1 & 2) and the concurrent surge in multiparous women with previous uterine scars (Robson Group 5) - are emblematic of a broader, global "triage effect" precipitated by the COVID-19 pandemic. Throughout the initial waves of the outbreak, stringent mobility restrictions, the reallocation of healthcare resources, and widespread patient apprehension regarding nosocomial SARS-CoV-2 transmission fundamentally altered obstetric care-seeking behaviors [2,9].

A comprehensive systematic review by Chmielewska et al. evaluating the global impact of COVID-19 on maternal outcomes demonstrated a precipitous decline in overall institutional deliveries, particularly in low- and middle-income countries (LMICs) [10]. Healthy women with uncomplicated, low-risk pregnancies frequently bypassed major metropolitan hospitals, opting instead for peripheral health centers, midwifery-led maternity units, or home births to distance themselves from the epicenters of the viral outbreak [1].

Consequently, tertiary care facilities were inadvertently transformed into high-acuity triage hubs. The concentration of clinical complexity observed in our study, characterized by older maternal age, higher parity, and a significantly heavier burden of metabolic comorbidities such as GDM, directly mirrors trends reported in international tertiary cohorts during the lockdown epochs. Patients presenting with a previous uterine section (Robson Group 5) or absolute surgical indications (Robson Groups 6-9) could not safely utilize peripheral centers and were funneled into the tertiary system, effectively saturating the admission profile with high-risk pregnancies [3,11].

This concentration of clinical complexity provides a critical epidemiological context for interpreting pandemic-era surgical rates. Early pandemic literature by Bhatia et al. [4] had reported alarming, unadjusted spikes in institutional cesarean delivery rates. However, our Robson-stratified analysis corroborates the emerging consensus that these initial reports were heavily influenced by a "denominator fallacy". The apparent surge in the crude surgical rate at tertiary centers was not driven by a diminished threshold for surgical intervention among obstetricians facing logistical panic; rather, it was a mathematical artifact caused by the disappearance of the denominator, the low-risk vaginal deliveries that had been diverted elsewhere [12,13]. By utilizing the Robson classification, our study unmasks this triage effect, confirming that, when adjusted for the shifting influx of surgical risk, institutional adherence to evidence-based delivery protocols remained remarkably resilient.

In our unadjusted analyses, the pandemic cohort's heavily concentrated burden of metabolic conditions, specifically GDM, chronic/gestational hypertension, and thyroid disorders, appeared to significantly increase the odds of surgical delivery. However, upon adjusting for maternal age and the Robson classification, these metabolic comorbidities completely lost their statistical significance.

This statistical attenuation highlights a well-documented epidemiological phenomenon: metabolic conditions frequently serve as proxy indicators for underlying demographic risk factors rather than acting as independent mandates for surgery [14,15]. Our adjusted model (aOR = 1.138; p = 0.174) aligns with recent global consensus demonstrating that uncomplicated GDM, when properly managed and in the absence of suspected fetal macrosomia, does not inherently justify a departure from vaginal delivery protocols [16].

The fact that these metabolic variables washed out in the multivariable model provides compelling evidence of clinical restraint. It indicates that the institution's obstetric department did not utilize the mere presence of a COVID-era, GDM, or hypertension diagnosis as a convenient threshold to expedite labor via surgery. Instead, surgical interventions were strictly reserved for cases where these metabolic conditions cascaded into acute, life-threatening complications or when they intersected with established obstetric risk factors.

By neutralizing the confounding effects of the pandemic's demographic shift, the regression model exposes the true, independent drivers of surgical delivery in our cohort. These drivers were rooted entirely in severe maternal morbidity and established obstetric indications, underscoring a rigorous adherence to global obstetric guidelines despite the logistical chaos of the COVID-19 lockdowns.

The most profound predictor of surgical delivery was the presence of a previous uterine scar (Robson Group 5), which increased the odds of a repeat LSCS more than 22-fold (aOR = 22.45). This finding is consistent with global data from the WHO, which consistently identifies Robson Group 5 as the single largest contributor to overall institutional cesarean rates worldwide, driven by the inherent risks of uterine rupture during a trial of labor after cesarean (TOLAC) [6,17]. Similarly, absolute indications, such as malpresentations, abnormal lies, and multiple gestations (Robson Groups 6-9), exhibited a nearly 11-fold increase in surgical odds, reflecting non-negotiable barriers to safe vaginal delivery [7].

Furthermore, while chronic hypertension lost its significance, preeclampsia and eclampsia remained massive independent risk factors, tripling the odds of surgical intervention (aOR = 3.05). This divergence perfectly illustrates evidence-based triage. Unlike chronic hypertension, severe preeclampsia represents an acute, progressive endothelial crisis where immediate, and often surgical, delivery remains the only definitive treatment to prevent maternal stroke, hepatic rupture, or fetal demise [7].

Further, the isolation of these specific, severe clinical determinants, advanced maternal age, previous surgical scars, malpresentations, and preeclampsia, confirms that the fundamental mechanisms of obstetric decision-making remained intact. The crude increase in surgical volume was a mathematical inevitability dictated by the triaged, high-risk physiology of the patients admitted, rather than a degradation of clinical practice standards by the attending physicians.

The primary strength of this study is its robust sample size (N = 3,656) and the integration of the WHO Robson classification with multivariable modeling, which successfully isolated true clinical drivers from pandemic-induced demographic confounders. However, as a single-center retrospective study, the findings may lack broad generalizability. Further, low-risk women who bypassed tertiary hospitals during lockdowns, meaning our dataset inherently reflects a highly triaged population. Additionally, unmeasured logistical confounders, such as acute staffing shortages or provider fatigue, could not be captured.

## Conclusions

The COVID-19 pandemic induced a profound "triage effect," fundamentally shifting the demographic profile of obstetric admissions toward older, multiparous, and highly comorbid patients. However, despite these surging clinical complexities and the unprecedented logistical pressures of the lockdown, delivering during the pandemic epoch did not independently increase a woman's odds of undergoing a cesarean delivery. Multivariable analysis confirms that surgical interventions remained strictly dictated by absolute, evidence-based obstetric indications - predominantly previous uterine scars, malpresentations, and severe preeclampsia. These findings highlight the remarkable resilience of the obstetric care system, demonstrating that rigorous clinical thresholds for surgical delivery were successfully maintained during a global public health crisis.
